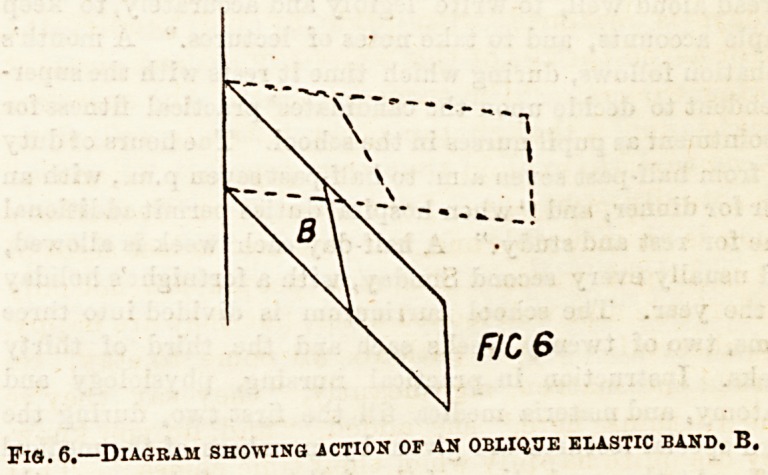# The Hospital Nursing Supplement

**Published:** 1895-09-21

**Authors:** 


### "The Hospital" Nursing Mirror.

Being the Extra Nursing Supplement of "The Hospital" Newspaper.

[Contributions for this Supplement should be addressed to the Editor, The Hospital, 428, Strand, London, W.C., and should have the word "Nursing" plainly written in left-hand top corner of the envelope.]

### News from the Nursing World.

### TAKING TEMPERATURES.

To a nurse accustomed to the diminutive clinical thermometer used in England, the long one in favour in French hospitals is a strange sight. The head nurse goes round the ward with a sheaf of tubes in her hand and places half-a-dozen of them with as many patients, with no fear of forgetting who "has the glass," for each of the latter is the length of an uncut pencil, and a portion is always visible. The stem is flattened out and the whole instrument is much stronger than the fragile little tubes which meet with such disastrous casualties in our wards.

### VOLUNTEERED APPROBATION.

"Mornin', nurse," said a workman, as a neat-looking district nurse got into the tram. She returned his greeting as she took her place in the crowded car. Presently she drew her hand from her pocket with an exclamation of dismay. "Oh! conductor, I've come without any money, and I can't go back because there's a poor woman waiting for me." The conductor was feeling cross, so he put up his hand to the bell. "I can't help that," he retorted; "you'll have to get out." Nurse looked distressed. "If you'll trust me," she said, "I promise to pay you this evening; I shall be coming this way again, and I will give you my address." But the conductor was obdurate, and nurse stood up bag in hand, ready to cry with vexation Suddenly the workman interposed. "Don't you do nothin' of the kind, nurse. I'll pay for your ticket; *I holds with midwives, I do.*"

### GRATEFUL PATIENTS.

The sick poor of Littleborough have shown their appreciation of the work done amongst them by Sister Lucie during the last four years, by bestowing on her several parting gifts and many good wishes. The presentation was made by Mrs. James Schofield at the Reform Club, and she spoke appreciatively of the successful lectures on nursing, which had been given by Sister Lucie under the auspices of the Littleborough Women's Liberal Association. A beautiful sewing machine, subscribed for by the members of the association, was amongst the useful presents which will remind Sister Lucie in her new district work in Cornwall of many friends who regret her departure from Lancashire.

### KIND AND CONSIDERATE.

"I wonder if you know of a tired nurse who would like to come and stay with me for a fortnight and enjoy rest and fresh air? She can use my library subscription and piano. If you send me anyone, please tell her that she will put herself under no obligation by coming; but, on the contrary, I shall be indebted to her for her companionship, as my life here is a quiet one." This pleasant letter was received by the Hon. Secretaries of The Hospital Convalescent Fund a few weeks ago, and the tempting invitation was promptly passed on to a nurse, who gratefully accepted it. At the end of her visit she wrote: "I never enjoyed anything more; my hostess seemed to feel she could hardly do enough for me. She made me at home at once, and her kindness and the change of air have made me better and stronger in all ways. I am exceedingly grateful for the care and consideration I received. . . ." Surely this excellent example is one which other ladies might follow.

### AN EXHIBITION OF NURSING APPLIANCES.

The forthcoming exhibition at the Trained Nurses' Club in Buckingham Street will possess special attractions for our readers. The editors of *Nursing Notes* are organising the exhibition, which is to consist of all kinds of nursing appliances, those invented by nurses being specially asked for. All exhibits must reach the club by October 5th, and it is expected that the collection will be thoroughly representative of the ingenuity of modern nurses.

### REGISTRATION IN SYDNEY.

From Sydney a correspondent writes: "A movement is on foot to establish some means whereby trained nurses may be registered, so that the many partially trained and, in some cases, untrained nurses may be known from those who are thoroughly competent. It is hoped that this will be carried out in its entirety, as there are many private nurses, established in some cases in so-called nurses' homes, who have never been inside a hospital wall in the capacity of nurse."

### AN ASYLUM ADVENTURE.

The tales we receive about the vagaries of officials are almost endless. It is not every day, however, that we come across so curious a story as that related in the *Poor Law Officers' Journal* about the removal of a pair of lunatics to an asylum near Yeovil. It appears that the relieving officer, while removing two female lunatics to the asylum, was accompanied by a female attendant. At the institution the relieving officer and the resident medical officer, after the usual formalities, left the three women together. One of the insane women was removed to the baths, and on the attendants returning for the other they, by mistake, seized upon the sane woman, and, despite her most energetic protests, screams, and the oft-repeated assertion that she was in full possession of her senses, she was forcibly taken to the baths, stripped, and bathed, the lunatic proper meanwhile being permitted to wander at will. Some little time after, on the relieving officer returning to take back with him the woman he had engaged as attendant to the lunatic, he was not a little astonished to find that she had been placed under restraint, while the lunatic was wandering about. No great harm was done, perhaps, but it is clear that if the relieving officer had left the attendant to find her own way home she might have been left in the asylum and the lunatic allowed at large.

### PRIZE-GIVING AT THE LONDON HOSPITAL.

The prizes gained by probationers of the London Hospital at the annual examination will be presented to them by Sir James Paget, Bart., F.R.S., D.C.L., on October 1st, in the library of the Medical College. The presentation of his portrait to Dr. J. Hughings Jackson, F.R.S., and the distribution of prizes to the students, will take place on the same occasion, when a large gathering of old "Londoners" is anticipated.

### A TRIBUTE TO SISTER AUGUSTINE.

During a recent visit paid by the President of the French Republic to the General Hospital at Langres an interesting little ceremony took place. A beautiful silver medal was presented to Sister Augustine on the completion by her of forty-five years of service. The President, in the course of a graceful little speech, acknowledged the valuable work which she had done amongst the sick and suffering poor.

### HELPING THE HELPLESS.

At a drawing-room meeting held in connection with the Ministering Children's League some time ago, the special claims on their sympathy of the Hospital and Home for Incurable Children in Maida Vale were brought to the notice of a large audience of young people. Boys and girls listened eagerly to anecdotes of the afflicted children, and they learnt what care they required and what great pleasures small kindnesses secured to these poor little people. Suggestions were made of various ways in which help could be given to further the good work with which all present seemed in sympathy. No doubt the speaker went away wondering whether any practical results would follow this appeal made for children to children. We have just learnt from Miss Coleman, the valued matron of the hospital and home, that she received in consequence of this appeal various contributions to the children's driving fund. A present of pinafores is also due to the drawing-room meeting. We, therefore, venture to hope that many more gifts may follow, and that the Ministering Children will not forget the old saw that "A little help is worth a deal o' pity."

### INCURABLE, BUT NOT INCONSOLABLE.

"And this is Grannie," said the Matron of St. Catherine's Home, at Bradford, as she opened the door of the pleasant day-room. Knowing that length of days is reckoned with pride by old people, the visitor asked the age of this white-capped and pleasant looking woman. "I'm turned seventy," she answered quickly, and although the other patients smiled, no one ventured to explain that this popular "Grannie" was actually eighty-five. Every case at St. Catherine's Home is an incurable one, but this fact is lost sight of in the bright wards. The sick people are not only comfortable and well looked after, but they are happy, nay, cheerful. "Our patients all come to stay," remarked the matron, significantly, "they say they've time to think here," she added, "and, do you know, they die quite happily when their time comes." That Miss Hadley Scott and the sister who works with her understand their patients goes without saying, and the latter are devoted to them both. "I can't die without matron," was the constant cry of one poor girl between paroxyms of terrible pain, and when, her short holiday ended, the matron came home and went straight to the girl's bedside, the end seemed very near. But she did not die after all, and is content to "live awhile longer, wi' our matron." Cancer, phthisis, heart disease are amongst the cases eligible for admittance to the home, and if the number of beds were doubled they would be all full. Unfortunately, one ward is unavailable, because there is at present no room for the extra nurse which its opening would necessitate. Those by whose liberality St. Catherine's Home was established are in constant touch with its inmates. They visit them frequently and bring books and presents for the patients, and keep every room supplied with flowers. The pall in the tiny mortuary is the work and gift of a Jewess, for the affection with which St. Catherine's is regarded is of a truly unsectarian character.

### SUPERSTITIOUS.

"Please, nurse, you're wanted!" "Who is it?" she inquired, rising from her seat. "They won't give me any message," answered the wardmaid, "but only say they must just see yourself for a minute." Two young factory girls stood at the door, and nurse invited them in, but they declined to sit down, though they had walked several miles at the end of a long day "at tha' mills." Mother had sent them, they explained, to make sure nurse was all right. Mother was "a rare one" for dreams, and she had dreamt about nurse last Friday, and they could not, any of them, feel satisfied without inquiring. The girls made light of the long walk entailed upon them by the influence of the dream, and went away cheerfully, convinced that on this occasion at any rate there was no ground for the fear that nurse was dead, though their parent had seen her "go off just like water down a ditch!"

### A HOSPITAL FOR INFANTILE DIARRHŒA.

Within half-an-hour's railway journey of Buffalo, in a charming situation at Athole Springs, there stands a most beautiful hospital. It contains every modern appliance to facilitate the skilled nursing of the sick, and it is expressly designed for the reception of babies suffering from serious forms of diarrhœa. Each infant is accompanied by its mother, or some other grown-up female relative, who remains with it until it is cured. There are resident physicians, a trained superintendent, and staff of nurses at the Buffalo Fresh Air Mission Hospital, and admirable results may be expected from their treatment, allied to hygienic surroundings, and wise dietary. Not only will new health and strength be gained for their little ones, but valuable lessons on the rearing of infants will be imbibed by the parents who share with them the advantages of this beneficent scheme.

### BRIDGWATER DISTRICT NURSE ASSOCIATION.

The second annual report of this association shows another successful year of work, and states that the patients have expressed themselves as most grateful for the kindness and attention shown them by Nurse Peterkin. It seems there are still funds in hand to carry on the work, but a considerable amount of what was in hand at the beginning of the year has been absorbed, and it is very necessary that the people in the district should maintain their interest in this institution, which, being in affiliation with the Queen Victoria Jubilee Institute, gives every guarantee of the efficiency of the nurses it employs. It is worth noting that a considerable proportion of the cases visited were under the care of parish doctors.

### Elementary Physiology for Nurses.

By C. F. MARSHALL, late Medical Registrar Hospital for Sick Children, Great Ormond Street.

### VII.—THE RESPIRATORY AND EXCRETORY SYSTEMS.

RESPIRATION consists of two chief factors: (1) Inspiration, effecting the introduction of oxygen. (2) Expiration, effecting the elimination of carbonic acid.

The general structure of a breathing organ is a moist vascular surface exposed to air or water; *e.g.*, the skin of a frog or the gills of a fish. In this way there is a direct interchange of gases, partly by chemical attraction and partly by physical diffusion. In the lower, and especially in smaller animals, the general surface of the body is sufficient for respiration, but in the higher animals special respiratory organs are required.

There are two chief plans by which respiration may be accomplished. One is to take the blood to some particular place, where it can be aerated; the other is to take air directly into all parts of the body, as is done in insects, by a system of air tubes permeating the body.

### RESPIRATORY ORGANS IN MAN.

These consist of the lungs and windpipe; the former being situated in the thorax and filling it, except for the heart and great blood vessels; the latter being placed in the neck. The trachea or windpipe ends above in the larynx or organ of voice. The trachea is kept open by cartilaginous rings in its walls, and below divides into two bronchi, one for each lung; these divide again and again till they open into little sacs, the air cells, in the substance of the lungs. The air cells are small sacs with very thin walls opening out of the smallest bronchial tubes, and in the thin septa between the adjacent air sacs the blood capillaries are exposed to air on each side. The pulmonary blood vessels form a fine network of thin-walled capillaries surrounding the air cells, like the network of string round a child's ball.

### THE MECHANISM OF RESPIRATION.

This is the means by which air is pumped in and out of the lungs. Let us first glance at the structure of the thorax or chest, which contains the organs of respiration. We find this to consist of a bony framework formed at the sides by the ribs, behind by the backbone, and in front by the sternum, or breast-bone. The base of the thorax is formed by the diaphragm, a muscular partition between the chest and abdomen.

The mechanism of respiration in man consists in the alternate expansion and contraction of the thoracic cavity. Everyone knows that in taking a deep inspiration the chest becomes inflated and expanded and increases in girth two inches or more. It is not that the air sucked in expands the chest, but that the chest expands and thereby sucks the air in. The expansion of the chest is of a double nature, partly costal, or due to the ribs; partly diaphragmatic.

1. *Diaphragmatic.*—The diaphragm is an arched dome-like muscle, tendinous in the centre and attached all round to the ribs, backbone, and sternum. Its contraction causes it to become more flattened, and hence enlarges the cavity of the chest, and diminishes the pressure of air in the lungs till it falls below the pressure of the atmospheric air. Air then rushes in through the windpipe to make up for this diminished pressure inside the chest.

2. *Costal.*—The ribs are curved rods of bone, movably joined behind to the vertebral columns and attached in front to the sternum. There are 12 ribs on each side, the upper ones being short. The first seven are attached to the sternum directly by separate cartilages; the next three indirectly by the cartilage of the seventh rib; the last two or floating ribs do not reach it at all. The ribs are directed downwards and forwards and not horizontally; on this fact their action depends.

Connecting the ribs together are the *intercostal muscles*, external and internal. The external intercostal muscles are directed downwards and forwards; the internal downwards and backwards. In action they may be compared to an oblique elastic band passing between the ribs: this will when contracting strive to assume the shortest position,

FIG. 5.—DIAGRAM OF THREE RIBS WITH INTERCOSTAL MUSCLES. E. External. I. Internal.

which will be when at right angles to the ribs. Hence when the intercostal muscles contract they must raise the ribs, as shown in the following diagrams. By this action the thorax is enlarged and air is sucked in. Both costal and diaphragmatic respiration go on simultaneously, the costal being more marked in women, the diaphragmatic in men.

Expiration is chiefly due to elastic recoil. The muscles cease to contract and the parts resume their former shape and position. This action is aided perhaps by the internal intercostal muscles as well. We must not suppose that the lungs are emptied of air, or anything like emptied at each expiration, or that air drawn in at each inspiration goes right into the air cells. In an ordinary inspiration 20 to 30 cubic inches of air enter the chest and pass out again at expiration. This is known as tidal air. Besides this a further amount can be taken in by forced inspiration, and an additional amount expelled by forced expiration. The former is known as complemental air, and may be 100 to 130 cubic inches; the latter is reserve or supplemental air, and is about 100 cubic inches. Besides this there is always a certain amount of residual air which cannot be got rid of after forced expiration. This is also about 100 cubic inches.

In normal or quiet breathing about 200 cubic inches—

FIG. 6.—DIAGRAM SHOWING ACTION OF AN OBLIQUE ELASTIC BAND. B.

residual and reserve air—are stationary, and about 20 to 30 cubic inches of tidal air are exchanged at each respiration. This tidal air will only get into the larger bronchial passages, and will certainly never reach the air cells directly. The interchange is effected by diffusion between the residual air in the lungs and the tidal air taken in at inspiration.

### New York City Training School for Nurses, Blackwell's Island.

ITS PAST HISTORY.

The twentieth annual report of this important New York training school is of a specially interesting character, for in it Miss Darche, whose name is well known in connection with nursing matters in America, prefaces her report as Superintendent with a review of the past history of the school. Originally started in 1875, in consequence of the recommendation of Dr. Kitchen, the Chief of Staff of Charity Hospital, at first there was no trained nurse appointed at its head, and "the Chief of Staff was left to perform the duties of training matron . . . as best he could." Before the close of the first year a change took place, but the Supervising Nurse appointed had a somewhat impossible position, for while responsibility as to discipline, conduct, and nursing efficiency rested upon her shoulders, the real authority was vested in a Board of Managers. The result was deterioration, and after a two years' struggle her resignation followed. It was at this point that Mrs. F. R. Jones, chairman of the State Charities Aid Visiting Committee to Charity Hospital, rose to the occasion. Her suggestions to the Commissioners were accepted, and she was herself empowered by them to select two capable women to fill the post of superintendent of the school and matron of the hospital, and Miss Darche and Miss Kimber were the candidates chosen. This was in 1888, and during the next two years the scope of the school became considerably increased. It is now responsible for the nursing of four distinct hospitals—the City, Maternity, Gouverneur, and Harlem Hospitals—"all under one central board of control, that of the Commissioners of Charities and Correction, but each having its own medical board, its own special methods of dealing with the sick, and each containing its own distinctive class of patients." Now the school numbers sixty-one nurses in training, and is controlled by a superintendent, assistant superintendent, and four supervising nurses. A school registry has been established on a co-operative basis, and is found to be of very material benefit to the nurses.

Miss Darche attributes the position held by the school to-day to the fact that it is managed upon the Merit system, and that the nurses are made to feel that "faithfulness to duty and integrity of character are the first and last essentials of her make up, that whatever else she may be, she is without these, as a nurse, valueless."

The Course of Training.

The course of training is for two years. During this period very complete instruction is given, practically and theoretically, in every branch of nursing. An entrance examination has to be passed "to test the applicants' ability to read aloud well, to write legibly and accurately, to keep simple accounts, and to take notes of lectures." A month's probation follows, during which time it rests with the superintendent to decide upon the candidates' practical fitness for appointment as pupil nurses in the school. The hours of duty are from half-past seven a.m. to half-past seven p.m., with an hour for dinner, and "when hospital duties permit additional time for rest and study." A half-day each week is allowed, and usually every second Sunday, with a fortnight's holiday in the year. The school curriculum is divided into three terms, two of twenty weeks each and the third of thirty weeks. Instruction in practical nursing, physiology and anatomy, and materia medica fill the first two, during the third special lectures are given by members of the medical staff. At the expiration of the full term of two years the nurses pass a final examination and receive a diploma of the school.

Male Training School.

A very important feature in connection with the New York City Training School is the experiment in the training of male nurses, which is being carried out under Miss Darche's direction. The course of training is for eighteen months. The requirements are as follows: Age, between twenty and thirty years, a good common school education, moral character, sound health, a willingness to learn and to work. Eligible candidates will be received for three months on probation. If at the end of this time they do not give satisfaction, their term of service is at an end; should they be accepted, it is included within the eighteen months' course. Their hours of duty in the hospital are from 6.40 a.m. to 6.30 p.m., with additional time off duty. Examinations have to be passed and instruction is given by the supervising nurse and the visiting physicians and surgeons. Miss Darche reports satisfactorily of the work accomplished. Since June of 1884 twenty-four young men have been admitted on probation, of whom eleven have been retained as satisfactory. Some of the best nurses on the male side are found to be the young men who intend making nursing a stepping-stone to the medical profession, and by these much good work is done. Since the organisation of the school eight years ago forty-six men have graduated; of these six are now practising medicine, five are at present attending medical schools, and twenty-six are nursing. These facts will probably be of special interest to many of our readers, to judge from the constant inquiries which reach us as to training of a similar kind in English hospitals. There is undoubtedly a great opening here which, it is much to be hoped, some hospital may soon be found sufficiently enterprising to take up and develop. The New York City Training School is certainly to be congratulated upon the work it is doing in the world of nursing.

### Appointments.

[It is requested that successful candidates will send a copy of their applications and testimonials, with date of election, to The Editor, The Lodge, Porchester Square, W.]

Bradford Children's Hospital.—Miss A. S. Woodhouse has been appointed Matron of this hospital. She was trained at the Worcester General Infirmary, and afterwards held the post of charge-nurse for three years in the women and children's wards of the infirmary at Bedford. For the last four and a half years Miss Woodhouse has held the position of charge-nurse at the Bedford Children's Hospital, where she has been universally liked and respected. She enters upon her new duties with many good wishes.

### Minor Appointments.

Derby County Asylum, Mickleover.—Miss Margaret Kirk has been made Chief Nurse at this asylum. She was trained at West Derby Union Hospital, and we congratulate her on her appointment.

Miss Maggie Morrison has been appointed Ward Sister to the City Hospital, Birmingham. She was trained at the King's Cross Fever Hospital, Dundee, and at the Salford Union Infirmary, where she is at present holding the position of theatre nurse and charge nurse of the male surgical pavilion, containing 100 beds. From both her posts she brings most excellent testimonials.

### "The Hospital" Convalescent Fund.

The Hon. Secretaries beg to acknowledge a subscription of 5s. from Nurse Talbot. Also £1 1s. from Mrs. Creighton Hale; 1s. 3d. from A. Thorpe (per Mr. Jenkins, male attendant); 5s. from Mrs. Cox and R. Barnes; 2s., Dr. Lloyd; 1s., Miss Wall; 1s., Miss Thomas; 1s., G. R. Jenkins. "I was very glad indeed to hear that 'The Bed' had been of so much use to many tired nurses, and I hope it will continue to be a comfort to many more," writes the male attendant, who has always given kindly help and encouragement.

### Everybody's Opinion.

[Correspondence on all subjects is invited, but we cannot in any way be responsible for the opinions expressed by our correspondents. No communications can be entertained if the name and address of the correspondent is not given, or unless one side of the paper only be written on.]

### GIRL LECTURERS.

DR. ALFRED T. SCHOFIELD writes: My attention has been called on my return from abroad to a remarkable paragraph in your issue for the 3rd ult., entitled "Girl Lectures." Girl lectures would appear at first sight an ungrammatical form of "Lectures to girls," but a perusal of the article reluctantly leads one to the supposition that the title was intended for "Girl Lecturers." The paragraph itself is entirely wrong in its gratuitous assumption that the lectures in question or even a majority of them are on nursing, and also fails entirely to point out the essential difference of the training requisite for a lady lecturer and a nurse. I also wish to say that these so-called "girls" are of the somewhat mature age of twenty-three before they can even commence their training. What the National Health Society has done, and I think wisely, is to recognise the difference between the training needed by those who have the actual care of patients in matters of life and death, as nurses, and ladies who give homely talks on physiology, hygiene, sanitation, home nursing, &c., by lowering the ages of the latter from twenty-six to twenty-four years. If the "girls," alas! are "cheap," that must be laid to the door of those county councils who do not sufficiently pay women's work. At any rate if cheap, the article is better, for the examination standard is always being raised.

[This matter is somewhat more important than Dr. Schofield seems to imagine. The age at which women can enter as probationers is twenty-five, the age of the National Health Society lecturers is to be twenty-four, and Dr. Schofield tells us that they must be twenty-three years old "before they can even commence their training." That is to say that, after only one year's training, they are to be considered competent to teach physiology, hygiene, sanitation, home nursing, &c., while this work of teaching is to be looked upon as of so little importance that it is to be entered upon as a way of profitably filling up the one year which intervenes before they can enter as probationers. We cannot too seriously urge upon County Councils and others interested in this matter that a lecturer should speak out of the fulness of her knowledge and experience, and should not merely disgorge what she has assimilated more or less successfully during her one year of training. Nothing can tend so much to cast ridicule on the art of nursing than that the very branch of it which demands the widest experience should be taught by those who are yet waiting to enter upon their practical training in its elements.—ED. T. H.]

### Novelties for Nurses.

### SICK-ROOM SHOES.

In private nursing something more homely and comfortable may often be adopted for the ease of tired feet than leather shoes, however soft and well fitting. For this purpose Messrs. Garrould have most neat and durable specialities. The shoes are made of dark felt, lined softly and warmly the soles, though pliant, have a durable sole which is conveniently roughened, so that there is no liability to slip. The shoes are most appropriate for the purpose for which they are intended, and are likely to be much appreciated.

### Nuns or Nurses.

RELIGION nowadays takes so many forms that one has almost ceased to be surprised at the odd things done in its name. The controversy, however, which has recently arisen at Athlone in regard to the employment of nuns as nurses cannot be passed over, even though the discussion may seem tainted with *odium theologium.* It appears that the Local Government Board have refused to sanction the appointment of Sister De Sales O'Connell as night nurse in the workhouse infirmary, on the ground that she is not a trained nurse. As the result of an inquiry held some time ago into the management of this infirmary, the Local Government Board, under threat of dissolving the board of guardians, required the latter to appoint a night nurse. The general nursing of the institution had hitherto been done by nuns, assisted by pauper wards-women; and without entering into the question of the charaeter of the nursing so performed, it is clear that the central Board would not have taken up such a strong position unless there had been considerable cause for dissatisfaction.

On this the guardians appointed Sister de Sales, and, to meet the wishes of the Local Government Board, assented to her undergoing a course of training in the Mater Misericordia Hospital, in Dublin, a plan to which, if our information is correct, Sister de Sales assented. The Local Government Board sanctioned this arrangement, and all seemed likely to go well; the poor were to have the benefit of improved nursing, and yet the Sisters were not to be displaced. It was under these circumstances that a most surprising religious difficulty is reported to have arison, the guardians having received a communication from the Bishop of Ardagh and Clonmacnoice stating that it was against the rules of the Order to which the nuns belonged to undergo training in the manner mentioned; and that if the guardians, as well as the Local Government Board, were not satisfied with the present experience of the nuns, he would withdraw them from the hospital. At a special meeting of the board of guardians the Local Government Board Inspector came before them with the object of getting them to alter the decision they had arrived at, and stated that, while he recognised the great influence which the nuns were for good in such an institution, it could not be contended that they were trained nurses, and the guardians had been directed to appoint a trained nurse.

After a long debate, however, the guardians decided that they would not appoint a trained nurse, but would retain the services of Sister de Sales, notwithstanding the threat to dissolve the board made by the Local Government Board.

From Athlone Sisters to Tottenham Deaconesses is a far cry, but it is clear that the same question is involved at both places. Put simply, the question is: Are not hospitals places for the cure of sickness? This has been answered harshly and ruthlessly in some countries; let it be answered here in the same sense, but more gently. Bishops, nuns, Sisters, Deaconesses, teetotalers, and vegetarians would all like to capture the hospitals for the propagation of their own faiths. Whatever else, however, they are allowed to do, they must not be permitted to lose sight of this elementary fact, that the first and predominant object of a hospital is the healing of the sick.

### Wants and Workers.

[THE attention of correspondents is directed to the fact that "Helps in Sickness and to Health" (Scientific Press, 428, Strand) will enable them promptly to find the most suitable accommodation for difficult or special cases.]

A LADY wants to know of a respectable home for a superior girl who is a good needlewoman. She suffers from heart disease, but is able to work. A small sum only could be paid for her.—*H. F. G.*

### For Reading to the Sick.

### SLEEPLESSNESS.

**Motto.**

Wearisome nights are appointed me.—*Job.*

**Verses.**

If in the night I sleepless lie,
My soul with heavenly thoughts supply.
—*Bishop Ken.*

Lord, a whole long day of pain
Now at last is o'er;
Ah! how much we can sustain
I have felt once more. . .

Could I face the coming night
If Thou wert not near?
Nay, without Thy love and might
I must sink with fear.
Round me falls the evening gloom,
Sights and sounds all cease,
But within this narrow room
Night will bring no peace.

Other weary eyes may close,
All things seek their sleep,
Hither comes no soft repose,
I must wake and weep.
Come, then, Jesus, o'er me bend,
Give me strength to cope
With my pains, and gently send
Thoughts of peace and hope.

Then if I must wake and weep
All the long night through,
Thou the watch with me wilt keep,
Friend and Guardian true;
In the darkness Thou wilt speak
Lovingly with me,
Though my heart may vainly seek
Words to breathe to Thee.
—*Lyra Germanica.*

Lines to be repeated by one who lies awake, the rhythm having a soothing effect:—

When courting slumber,
The hours I number,
And sad thoughts cumber
My weary mind;
The thought doth cheer me
That Thou art near me,
Whose ear to hear me
Is well inclined.

My soul Thou keepest,
Who never sleepest,
Midst gloom the deepest
There's light above.
Thine arm enfolds me,
Thy grace upholds me,
Thy word has told me
That "God is love."—*The Christian.*

**Reading.**

Did you ever think that the words, "Wearisome nights are appointed me," carry with them the truest comfort? They do not come by any chance or accident then. God ordered them for you. He knows how many such you need, and He will not give you one more than is necessary. Surely in His own last night of agony He tasted the extreme of your suffering, and when He sends you the sharpest pains, so that your "whole head is sick and your heart faint" . . . . even then He is by, Who in wonderful pity and condescension has promised to "make all your bed in your sickness." . . . Think when you are able of His unspeakable nearness to you, that the darkness hideth not from Him, but the night shineth as the day; the darkness and the light to Him are both alike. You cannot pray perhaps continuously; but however short the petition may be, even a mere groan, it will reach His ear. Lay yourself down then quietly in His arms, and believe that the Eternal God is thy refuge, and underneath are the everlasting arms, then "thou shalt not be afraid of any terror by night," for "the Lord is thy reward."
—*Sickness: Its Trials and Blessings.*

### Where to Go.

Exhibition of Old China and Glass at the Westminster Aquarium.—Taken as a whole, this exhibition is of an educative value and not alone "a pleasure to the eye"; it is both beautiful and instructive. Here we see, side by side, ancient and modern china, the old and the new, in many very varied specimens, and in the natural order of events we fall to criticising them. Such comparison must, perhaps, necessarily be a purely personal matter, but we are inclined, anyhow in the present case, to give to the British fabrication a greater share of praise than to any other. Here it is placed in immediate proximity to the beautiful work turned out from Dresden and Sèvres, and certainly it shows to no mean advantage amongst any of the foreign art collected here. Amidst so much of interest, it is not easy to single out any particular objects from the general wholes, but the stall of Messrs. Stonor and Evans is perhaps the most striking; it is full of beautiful and unique specimens. Here there is rare old Worcester, old Bristol, and Staffordshire china. Amongst the old Worcester is Lord Nelson's celebrated service. Then, too, there are here a perfect bevy of little patch boxes belonging to dead and gone beauties, who have long since passed away. Some of these relics have quaint old-world mottoes painted in on the china; but there are also other treasures too innumerable to individualise. It is to the china more than to the glass that we turn our attention at the present exhibition, because the former is rather more to the fore, but there is some Venetian glass shown by the Venice and Murano Company, which is of extremely fine and beautiful workmanship. The organisers of the loan collection are to be congratulated on the success of their endeavours, and the exhibition is one which most fully repays a visit.

### Notes and Queries.

The contents of the Editor's Letter-box have now reached such unwieldy proportions that it has become necessary to establish a hard and fast rule regarding Answers to Correspondents. In future, all questions requiring replies will continue to be answered in this column without any fee. If an answer is required by letter, a fee of half-a-crown must be enclosed with the note containing the enquiry. We are always pleased to help our numerous correspondents to the fullest extent, and we can trust them to sympathise in the overwhelming amount of writing which makes the new rules a necessity. Every communication must be accompanied by the writer's name and address, otherwise it will receive no attention.

**Queries.**

(249) *Male Nurses.*—Is there any hospital in England where men can be trained as nurses?—*I. I. W.*

(250) *Maternity Nurse.*—Can you tell me if there is any hospital or infirmary where a certificated maternity nurse, aged 40, could receive training in medical and surgical nursing? Small premium could be paid.

**Answers.**

(249) *Male Nurses.*—No English hospital has as yet made arrangements for giving training to men. Inquire at the Seamen's Hospital, Greenwich.

(250) *Maternity Nurse.*—Write to lady superintendents of several hospitals. Complete list of London and provincial hospitals will be found in Burdett's "Hospital and Charities Annual," 428, Strand, London, W.C.

## Figures and Tables

**Fig. 5 f1:**
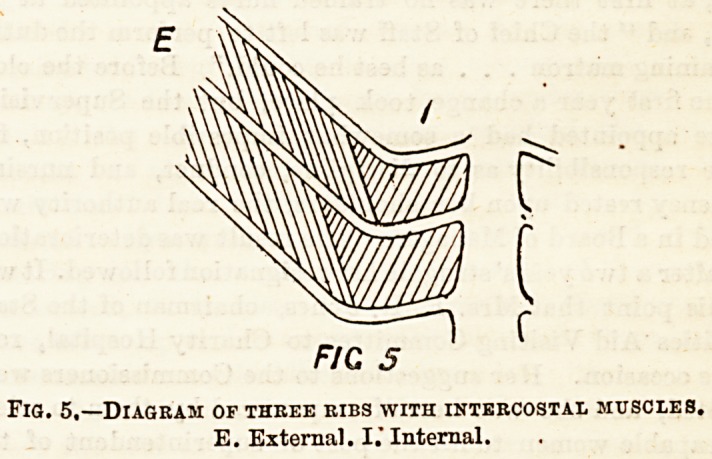


**Fig. 6 f2:**